# Adaptive Local Spatiotemporal Features from RGB-D Data for One-Shot Learning Gesture Recognition

**DOI:** 10.3390/s16122171

**Published:** 2016-12-17

**Authors:** Jia Lin, Xiaogang Ruan, Naigong Yu, Yee-Hong Yang

**Affiliations:** 1 Faculty of Information Technology, Beijing University of Technology, Beijing 100124, China; adrxg@bjut.edu.cn (X.R.); yunaigong@bjut.edu.cn (N.Y.); 2 Beijing Key Laboratory of Computational Intelligence and Intelligent System, Beijing 100124, China; 3 Department of Computing Science, University of Alberta, Edmonton, AB T6G2E8, Canada; herberty@ualberta.ca

**Keywords:** gesture recognition, one-shot learning, spatiotemporal feature, adaptive, optical flow, motion region of interest

## Abstract

Noise and constant empirical motion constraints affect the extraction of distinctive spatiotemporal features from one or a few samples per gesture class. To tackle these problems, an adaptive local spatiotemporal feature (ALSTF) using fused RGB-D data is proposed. First, motion regions of interest (MRoIs) are adaptively extracted using grayscale and depth velocity variance information to greatly reduce the impact of noise. Then, corners are used as keypoints if their depth, and velocities of grayscale and of depth meet several adaptive local constraints in each MRoI. With further filtering of noise, an accurate and sufficient number of keypoints is obtained within the desired moving body parts (MBPs). Finally, four kinds of multiple descriptors are calculated and combined in extended gradient and motion spaces to represent the appearance and motion features of gestures. The experimental results on the ChaLearn gesture, CAD-60 and MSRDailyActivity3D datasets demonstrate that the proposed feature achieves higher performance compared with published state-of-the-art approaches under the one-shot learning setting and comparable accuracy under the leave-one-out cross validation.

## 1. Introduction

Vision-based gesture recognition is a critical interface for non-intrusive human-robot interaction (HRI) systems, for which many natural and convenient recognition methods have been proposed [1,2,3]. In a traditional HRI system, a user usually needs to memorize a predefined gesture language or a set of instructions before interaction. Although users can learn new gesture motions, they may not enjoy learning the predefined gesture language or instructions. In particular, when a user plans to define and use arbitrary interaction gestures, existing HRI systems require labour-intensive and time-consuming procedures to collect and label all the training samples. This is because most of the traditional methods based on supervised learning require many training samples and long training times. All of these factors render a typical HRI system unsuitable for adding user-defined gestures easily and hence, motivate the current research of one-shot learning gesture recognition in solving the above mentioned problems.

One-shot learning is a concept cognition and learning method that models after a human’s innate learning ability. For example, humans can learn and generalize a new concept from one or very few samples [4]. Unlike tradition methods, one-shot learning gesture recognition is a small sample size learning problem, which means that every gesture has only one or very few training samples [5,6,7,8,9]. When learning a new gesture, the user needs to perform the gesture only once or a few times without the need to collect a large number of training samples. Meanwhile, simple classification methods, which do not need a lot of time for offline learning, can satisfy well the identification requirements. One-shot learning gesture recognition not only greatly improves the ease-of-use of HRI, but also makes the robot learn and recognize interaction gestures with the cognition mechanism conforming to the expectation of a human user.

One important challenge of one-shot learning gesture recognition is to accurately extract distinctive features from a small number of training samples. These features should represent well the between-class differences and within-class similarities of gestures [1,5]. Some recently published approaches use the color or depth information to detect spatiotemporal interest points (STIPs), i.e., keypoints, from which to build the feature descriptors. The spatiotemporal feature approaches (keypoints + feature descriptors) do not need any preprocessing steps, such as human detection, segmentation or skeleton extraction. Hence, they are highly flexible for applying to different environments. In fact, a user can perform gestures in 3D space. If the features are extracted from a single channel (color or depth), the loss of information in the other channel will greatly reduce the representative and discriminative ability of gesture features, so the study of RGB-D data-based spatiotemporal feature approaches has received much attention from researchers.

Hernandez-Vela et al. [10,11] detected keypoints using the Harris 3D detector [12,13] on RGB and depth volumes. Then, Histogram of Oriented Gradients (HOG) [14], Histogram of Optical Flow (HOF) [15], HOG/HOF [15] and Viewpoint Feature Histogram Camera Roll Histogram (VFHCRH) feature descriptors are calculated for each keypoint. The 3D motion scale invariant feature transform (3D MoSIFT) [16], 3D enhanced MoSIFT (3D EMoSIFT) [6] and 3D sparse MoSIFT (3D SMoSIFT) [1] spatiotemporal feature approaches, which are extensions of the MoSIFT method [17] using RGB-D data, can be referred to as 3D MoSIFT-based methods. All the 3D MoSIFT-based methods detect the initial interest points in whole frames. Then, these interest points of 3D MoSIFT whose grayscale velocities satisfy certain motion constraints are treated as keypoints. 3D EMoSIFT increases the depth-dependent constraints on the basis of 3D MoSIFT. 3D SMoSIFT extracts keypoints from the initial interest points by applying the grayscale or depth absolute velocity constraints, which are constant and determined by trial-and-error. Finally, the 3D MoSIFT-based methods use fused RGB-D data to construct SIFT-like feature descriptors. The Mixed Features Around Sparse Keypoints (MFSK) method [9] uses a strategy similar to that of 3D SMoSIFT to detect keypoints. The difference between them is that the MFSK method [9] detects Speeded Up Robust Features (SURF) corners [18] as initial interest points while 3D SMoSIFT uses Shi-Tomasi corners [19]. Then 3D SMoSIFT, HOG, HOF and motion boundary histogram (MBH) feature descriptors [20] are calculated from the RGB-D data. These one-shot learning gesture recognition approaches have achieved good performance using the 2011/2012 ChaLearn One-shot-learning Gesture Dataset (CGD) [21].

Besides the motion caused by the moving body parts (MBPs), motion may also exist in the background and the remaining body parts (non-MBPs). These motions may be caused by illumination changes, moving shadows, inaccurate or lost depth values (black block in a depth frame) or clothes movements (moving together with the MBPs), all of which can be collectively referred to as noise. The above mentioned spatiotemporal feature approaches directly detect keypoints in the whole frame, according to some predefined empirical motion constraints, which can incorrectly label some noise points with larger motion as keypoints. Because the speeds of different MBPs are not the same, constant motion constraints cannot guarantee correct detection of accurate and a sufficient number of keypoints in every MBP. If the constraint thresholds were set too high, features with relatively small movements would be ignored. On the contrary, if the constraint thresholds were set too low, many noise points would be detected. Different users or even the same user performing the same gesture at different times may exhibit different speeds and also the actual hardware execution environment of HRI varies. Hence, constant empirical motion constraints will not work in all situations.

To address the above problems, an adaptive local spatiotemporal feature (ALSTF) which combines both the appearance and the motion information is proposed. First, variances of grayscale and depth optical flows are used to adaptively extract motion regions of interest (MRoIs) which mainly contain MBPs, and to remove regions of the background and of the remaining body parts which contain a lot of noise. Then, Harris-Affine corners [22] are used as the initial interest points in MRoIs. The initial interest points with depth values and velocities of grayscale and depth that satisfy new local adaptively determined motion constraints in each MRoI, are regarded as the keypoints. Finally, multi-feature descriptors, computed in the new extended gradient and motion spaces, are combined to represent gestures. The major contributions of our new method include:

- A new adaptive MRoIs extraction approach is proposed to reduce the effect of noise on the accuracy of extracting spatiotemporal features.
- In each MRoI, new local depth and motion constraints are adaptively determined to detect keypoints. By this, not only the influence of noise can be reduced, but also the accuracy of detecting keypoints can be improved and more features can be extracted in the MBPs with large speed differences.
- 3D SMoSIFT, HOG, HOF and MBH feature descriptors are calculated in the new extended gradient space and motion space, and employed to generate more RGB–D appearance and motion features of gestures.
- Compared with other spatiotemporal feature approaches and with the published one-shot learning gesture recognition approaches, the proposed method achieves a better recognition performance.

The rest of this paper is organized as follows: Section 2 briefly reviews related works on spatiotemporal feature approaches. Section 3, Section 4 and Section 5 describe details of the proposed method. The adaptive MRoIs extraction approach is presented in Section 3.1; Section 3.2 shows how to adaptively detect keypoints in each MRoI. The improved feature descriptor calculation process is illustrated in Section 4. Section 5 introduces a resolution strategy for a special case wherein the human body is maintained in a relatively static condition during one stage of a gesture. Then the experimental results are shown in Section 6, where the evaluations and comparisons with state-of-the-art algorithms are discussed. Finally, Section 7 concludes this paper and includes some discussions on future work.

## 2. Related Works

Spatiotemporal feature approaches belong to the single-layered human gesture recognition approach. They consider a gesture as a set of appropriate features extracted from a 3D space-time volume and recognize the gesture from an unknown video sequence by categorizing it into one of the known classes [23]. In the following, some spatiotemporal features used in state-of-the-art techniques on gesture recognition and one-shot learning gesture recognition tasks are described.

Laptev et al. [12,13] proposed the Spatiotemporal Interest Point (STIP) detector, i.e., Harris 3D corner detector, to detect interest points in the spatiotemporal domain. The Harris 3D detector is a spatiotemporal extension of the Harris corner detector [24]. First, a spatiotemporal second-moment matrix μ and a normalized Laplace operator $\nabla_{norm}^{2}L$ of each grayscale pixel are computed. The grayscale points with local positive maxima of the corner function $H=\det(\mu )\;-\;k{\mathrm{trace}}^{3}(\mu )$ and extrema of the operator $\nabla_{norm}^{2}L$ are regarded as STIPs. Then, local, spatiotemporal and scale-invariant N-jet descriptors are used to represent and classify events of interest.

The Cuboid [25] feature uses a new response function for the Harris 3D detector to detect STIPs. Its response function is composed of a 2D Gaussian smoothing kernel for the spatial dimensions and a quadrature pair of 1D Gabor filters for the temporal dimension. The locations of interest points are given by the local maxima of the response function. Once detected, the cuboid around each interest point, which contains the pixel appearance values of the interest point’s neighborhoods, is extracted [23]. By testing various transformations to be applied to cuboids to extract the final local features, the flattened vector of brightness gradients with the best performance is chosen as the descriptor, which is essentially a generalization of the Principal Components Analysis-Scale Invariant Feature Transform (PCA-SIFT) descriptor [23,25].

Lu et al. [26] employed spatiotemporal filtering and noise suppression to construct the response function in the spatiotemporal and scale domain from depth videos. Depth STIPs (DSTIPs) are selected at the local maxima of the response function. The Depth Cuboid similarity feature (DCSF) descriptor is used to encode the spatiotemporal appearances of the 3D cuboid around the DSTIP, based on self-similarity.

The Hessian detector [27] is an extension of the Hessian saliency measure [28,29] for blob detection in the spatiotemporal domain. The localization and scale selection of interest points are directly implemented and selected, without iteration, by measuring saliency with the determinant of the 3D Hessian matrix. To describe interest points, an extended version of the SURF descriptor is proposed. Descriptors are calculated in both spatial and temporal domains simultaneously. For rotation invariance, the dominant orientation is used in the spatiotemporal domain. Meanwhile, all the Haar-wavelets are extended over the full length of the temporal scale of the interest point [27].

Wang et al. [30] proposed representing human actions using dense trajectories and motion boundary descriptors. First, feature points are densely sampled on a grid space and the Shi-Tomasi detector [19] is used to remove points in the image areas that have no structure. Then, each feature point is tracked over L frames to form a trajectory. Finally, a trajectory shape descriptor is employed to encode local motion patterns of actions. Besides, a concatenated feature descriptor of HOG, HOF and MBH is used to represent the appearance and motion information.

Chen et al. [17] proposed the MoSIFT method, based on RGB and optical flow information. Since it is derived from SIFT [31], it is scale and rotation invariant. First, the Gaussian pyramids and the Difference of Gaussian (DoG) pyramid are constructed for two consecutive grayscale frames. The local extrema of the DoG pyramid are selected as the initial interest points. Then, the optical flow pyramids corresponding to the grayscale pyramids are calculated, and for each interest point to be a keypoint, its grayscale optical flow must satisfy certain empirical motion constraint thresholds. Finally, the MoSIFT feature descriptors of the keypoints are computed using the grayscale Gaussian pyramid and the optical flow pyramids.

Hernandez-Vela et al. [10,11] used the Harris 3D detector to detect keypoints $S_{RGB}$ in the RGB volumes and keypoints $S_{D}$ in the depth volumes. Then, the HOG, HOF and HOG/HOF feature descriptors for $S_{RGB}$ and the VFHCRH descriptors for $S_{D}$ are calculated to represent gestures. This approach is an extension of the Harris 3D detector using RGB and depth data.

3D MoSIFT [16], 3D EMoSIFT [6] and 3D SMoSIFT [1] are derived from MoSIFT using RGB-D data. 3D MoSIFT and 3D EMoSIFT adopt a similar strategy to detect initial interest points. 3D SMoSIFT just detects the Shi-Tomasi corners in grayscale scale space as initial interest points to speed up processing. To extract keypoints from the initial interest points, different 3D MoSIFT-based methods have their own individual strategies. In particular, 3D MoSIFT takes the grayscale motion constraints which are the same as that used in MoSIFT. On the basis of MoSIFT and 3D MoSIFT methods, 3D EMoSIFT includes a depth-dependent constraint to remove some noise points. 3D SMoSIFT adopts grayscale or depth motion constraints to extract keypoints. The difference between 3D MoSIFT and 3D SMoSIFT is that 3D SMoSIFT uses the magnitude of the velocity while 3D MoSIFT uses the magnitudes of the horizontal and vertical velocity components. After keypoints are detected, 3D MoSIFT-based methods construct a 3D gradient space and a 3D motion space to calculate SIFT-like feature descriptors in these spaces. They adopt the same 3D gradient space which are constructed using the grayscale and depth spatial information. The temporal variations of the grayscale optical flow and of the depth information are employed for constructing a 3D motion space for 3D MoSIFT and 3D EMoSIFT. 3D SMoSIFT improves the 3D motion space by simultaneously using the grayscale and the depth optical flow simultaneously.

The MFSK [9] spatiotemporal features are derived from 3D SMoSIFT. The difference between the two is that, for detecting the initial interest points, MFSK uses the SURF detector while 3D SMoSIFT employs the Shi-Tomasi corner detector; on the basis of 3D SMoSIFT feature descriptors, MFSK includes the HOG, HOF, and MBH feature descriptors, which can well represent the appearance and motion features of gestures.

It can be inferred that the above feature-based approaches detect keypoints from whole frames in the spatiotemporal domain. Some approaches rely on grayscale or depth cues to detect keypoints, which include keypoints due to noise. Although other approaches add motion information to filter out noise, constant global empirical motion constraints are not very good to adapt to various possible scenarios. Therefore, the adaptive local spatiotemporal feature is worth studying.

## 3. Keypoint Detection

Firstly, to reduce the effect of noise, MRoIs are adaptively extracted on the basis of variance information of grayscale and depth optical flows. Then, a sufficient number of accurate keypoints are detected using adaptive local motion and depth constraints in each MRoI.

**Figure 1:**
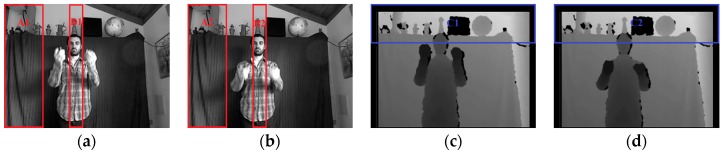
Two pairs of consecutive gesture frames captured at time t and t+1. (**a**) Grayscale frame $G_{t}$; (**b**) Grayscale frame $G_{t+1}$; (**c**) Depth frame $D_{t}$; (**d**) Depth frame $D_{t+1}$. Noise exists in regions A1, A2, B1, B2, C1 and C2.

To introduce the proposed approach more intuitively, two pairs of consecutive frames are used to illustrate every stage, as shown in Figure 1. The grayscale frames $G_{t}$, $G_{t+1}$ (converted from RGB frames) and the depth frames $D_{t}$, $D_{t+1}$ are captured by Kinect at time t and t+1, respectively. The frame resolution is N×M, where M is the number of rows, and N the number of columns.

### 3.1. Adaptive MRoIs Extraction

**Figure 2:**
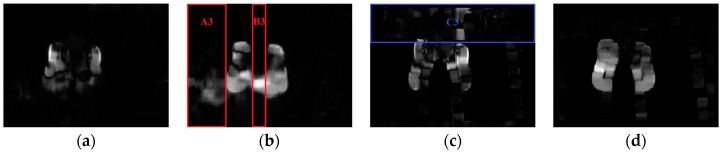
Grayscale and depth optical flow corresponding to Figure 1. (**a**) Grayscale horizontal velocity $V_{G}^{t,r}$; (**b**) Grayscale vertical velocity $V_{G}^{t,c}$; (**c**) Depth horizontal velocity $V_{D}^{t,r}$; (**d**) Depth vertical velocity $V_{D}^{t,c}$.

The Farneback algorithm [32] is adopted to obtain dense grayscale (depth) optical flow of $G_{t}$, $G_{t+1}$ ($D_{t}$, $D_{t+1}$). The grayscale (depth) optical flow consists of the horizontal velocity $V_{G}^{t,r}$ ($V_{D}^{t,r}$) and the vertical velocity $V_{G}^{t,c}$ ($V_{D}^{t,c}$), as illustrated in Figure 2. Then, variance vectors $S_{G}^{t,r}=\left\{s_{G}^{t,r,1},\ldots ,s_{G}^{t,r,i},\ldots ,s_{G}^{t,r,M}\right\}$, $S_{D}^{t,r}=\left\{s_{D}^{t,r,1},\ldots ,s_{D}^{t,r,i},\ldots ,s_{D}^{t,r,M}\right\}$, $S_{G}^{t,c}=\left\{s_{G}^{t,c,1},\ldots ,s_{G}^{t,c,j},\ldots ,s_{G}^{t,c,N}\right\}$ and $S_{D}^{t,c}=\left\{s_{D}^{t,c,1},\ldots ,s_{D}^{t,c,j},\ldots ,s_{D}^{t,c,N}\right\}$ are calculated, respectively. $s_{G}^{t,r,i}$ and $s_{D}^{t,r,i}$ ($s_{G}^{t,c,j}$ and $s_{D}^{t,c,j}$) are the magnitude variances of the *i*th row (*j*th column) of $V_{G}^{t,r}$ and $V_{D}^{t,r}$ ($V_{G}^{t,c}$ and $V_{D}^{t,c}$), respectively.

**Figure 3:**
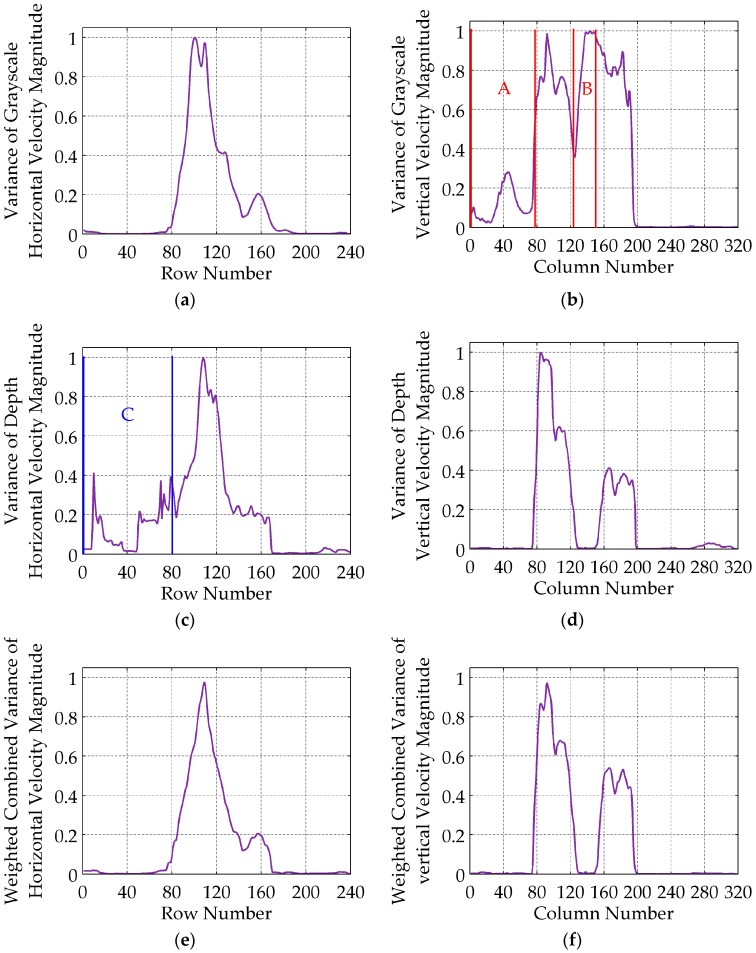
Row and column variance vectors of grayscale and depth velocity magnitude. (**a**) Row variance vector $S_{G}^{t,r}$ of grayscale horizontal velocity magnitude; (**b**) Column variance vector $S_{G}^{t,c}$ of grayscale vertical velocity magnitude; (**c**) Row variance vector $S_{D}^{t,r}$ of depth horizontal velocity magnitude; (**d**) Column variance vector $S_{D}^{t,c}$ of depth vertical velocity magnitude; (**e**) Weighted combined row variance vector $S_{G,D}^{t,r}$ of horizontal velocity magnitude; (**f**) Weighted combined column variance vector $S_{G,D}^{t,c}$ of vertical velocity magnitude. $S_{G}^{t,r}$, $S_{G}^{t,c}$, $S_{D}^{t,r}$ and $S_{D}^{t,c}$ are normalized.

When $s_{G}^{t,r,i}$ or $s_{D}^{t,r,i}$ ($s_{G}^{t,c,j}$ or $s_{D}^{t,c,j}$) is larger, the corresponding *i*th row (*j*th column) of $G_{t}$ or $D_{t}$ has a faster horizontal (vertical) motion. If a row (column) interval of $G_{t}$ or $D_{t}$ contains large horizontal (vertical) motions, all the $s_{G}^{t,r,i}$ or $s_{D}^{t,r,i}$ ($s_{G}^{t,c,j}$ or $s_{D}^{t,c,j}$) of this interval are larger. These motions are not caused by gesture alone, but noise as well. For example, there are relatively large $s_{G}^{t,c,j}$ in intervals A and B (Figure 3b), which are caused by the obvious vertical motions of shadow in regions A1, A2 and B1, B2 (Figure 1a,b), respectively. The corresponding grayscale vertical velocities are shown in regions A3 and B3 of Figure 2b. The larger depth horizontal velocities in region C3 (Figure 2c) are caused by the loss of depth values in regions C1 and C2 (Figure 1c,d), and shown as row interval C in Figure 3c, in which all the $s_{D}^{t,r,i}$ are larger. If keypoints are detected within the whole frame according to constant empirical motion constraints, as the 3D MoSIFT-based and MFSK methods, numerous noise points will be falsely detected as keypoints in the larger motion regions.

Usually, the motion of MBPs would cause changes in both grayscale and depth. But the depth of regions with moving shadow or illumination change remain nearly unchanged; the grayscale of regions with larger motion caused by depth value loss are mostly stable. Although clothes movements would cause changes in grayscale and depth, the changes of these regions are generally small. So, the grayscale and depth velocity magnitude variances are combined to extract MRoIs by eliminating noise to the extent possible.

After normalizing $S_{G}^{t,r}$ and $S_{D}^{t,r}$, the weighted combined vector $S_{G,D}^{t,r}$ of the $S_{G}^{t,r}$ and $S_{D}^{t,r}$ is given by:

(1) $$S_{G,D}^{t,r}=\left\{s_{G,D}^{t,r,i}|s_{G,D}^{t,r,i}=k_{1}\exp(s_{G}^{t,r,i})+(1-k_{1})\;\exp(s_{D}^{t,r,i}),\;1\leq i\leq M\right\},$$

where $k_{1}=s_{D}^{t,r,i}/\left(s_{G}^{t,r,i}+s_{D}^{t,r,i}\right)$ is the weight of $\exp(s_{G}^{t,r,i})$, then:

(2) $$s_{G,D}^{t,r,i}=\frac{s_{D}^{t,r,i}\exp(s_{G}^{t,r,i})+s_{G}^{t,r,i}\exp(s_{D}^{t,r,i})}{s_{G}^{t,r,i}+s_{D}^{t,r,i}}.$$

If the *i*th row of $G_{t}$ or $D_{t}$ contains a larger horizontal noise motion, then one of $s_{G}^{t,r,i}$ or $s_{D}^{t,r,i}$ is much larger than the other. To reduce the influence of noise, a small weighted value $s_{G,D}^{t,r,i}$ can be obtained by Equations (1) and (2) where a smaller weight is assigned to the larger magnitude variance and vice versa. But when $s_{G}^{t,r,i}$ and $s_{D}^{t,r,i}$ are both larger or smaller, the combined $s_{G,D}^{t,r,i}$ varies similarly. The weighted combined vector $S_{G,D}^{t,c}$ of the normalized $S_{G}^{t,c}$ and $S_{D}^{t,c}$ is calculated according to Equation (3):

(3) $$S_{G,D}^{t,c}=\left\{s_{G,D}^{t,c,j}|s_{G,D}^{t,c,j}=\frac{s_{D}^{t,c,j}\exp(s_{G}^{t,c,j})+s_{G}^{t,c,i}\exp(s_{D}^{t,c,j})}{s_{G}^{t,c,j}+s_{D}^{t,c,j}},\;1\leq j\leq N\right\}.$$

The weighted combination can well suppress the larger variances caused by noise, but preserve the large variances intervals including MBPs, as shown in Figure 3e,f. The $S_{G,D}^{t,r}$ and $S_{G,D}^{t,c}$ are normalized.

Accordingly, the *i*th row or the *j*th column of $G_{t}$ and $D_{t}$ can be treated as a part of the horizontal or vertical MRoI, while $s_{G,D}^{t,r,i}$ or $s_{G,D}^{t,c,j}$ satisfies constraints (4) or (5):

(4) $$s_{G,D}^{t,r,i}\geq \alpha_{1},1\leq i\leq M,$$

(5) $$s_{G,D}^{t,c,j}\geq \alpha_{2},1\leq j\leq N,$$

where $\alpha_{1}$ and $\alpha_{2}$ are adaptively determined from $S_{G,D}^{t,r}$ and $S_{G,D}^{t,c}$, respectively. $\alpha_{1}$ can be obtained using the following steps: (1) Calculate the binarization threshold $th_{1}$ of $S_{G,D}^{t,r}$ according to the Otsu algorithm [33], and the elements of $S_{G,D}^{t,r}$ with values less than $th_{1}$ constitute a set $H_{1}$; (2) Calculate the threshold $th_{2}$ of $H_{1}$ also according to the Otsu algorithm, then $\alpha_{1}=th_{2}$. $\alpha_{2}$ is calculated according to the same method.

**Figure 4:**
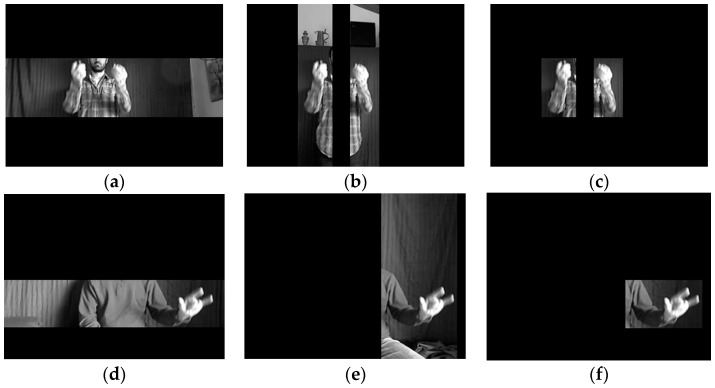
Extracted horizontal, vertical and final MRoIs. (**a**–**c**) The MRoIs of two moving hands frame; (**d**–**f**) The MRoIs of single moving hands frame. (**a**,**d**) The horizontal MRoIs; (**b**,**e**) The vertical MRoIs; (**c**,**f**) The final MRoIs.

The intersections of the horizontal and the vertical MRoIs are the final MRoIs. As indicated in Figure 4, the extracted MRoIs contain complete MBPs, while the background and the non-MBPs are removed effectively, so the next step is focused on detecting keypoints in each MRoI.

### 3.2. Adaptive Keypoint Detection

For one-shot learning gesture recognition, the limited number of training samples requires that the detected keypoints are affine invariant, besides being scale, rotation and illumination invariant. The Harris-Affine corners are chosen as the candidate keypoints. Using the Harris-Affine corners can potentially increase the number of keypoints, which can improve the quality of the representation of the hand appearance and of its shape.

To detect Harris-Affine corners, a Gaussian scale space is constructed first. The grayscale and depth Gaussian scale spaces $P_{G}^{t}$, $P_{D}^{t}$ are constructed using Equation (6) at time t [22]:

(6) $$P_{G}^{t,l}=g(k^{l-1}\sigma )*G_{t},1\leq l\leq L,\;P_{D}^{t,l}=g(k^{l-1}\sigma )*D_{t},1\leq l\leq L,$$

where L is the number of levels of scale space; $P_{G}^{t,l}$ and $P_{D}^{t,l}$ are the $l^{th}$ level of $P_{G}^{t}$ and $P_{D}^{t}$, respectively; k is a scale factor; σ is an initial smoothing parameter of the Gaussian function g(⋅); and ∗ is the convolution operator. At time t+1, the Gaussian scale spaces $P_{G}^{t+1}$, $P_{D}^{t+1}$ for $G_{t+1}$ and $D_{t+1}$ are also constructed using Equation (6). $P_{G}^{t}$, $P_{D}^{t}$, $P_{G}^{t+1}$ and $P_{D}^{t+1}$ together form the final Gaussian scale space. Although the method of extracting MRoIs from $G_{t}$ and $D_{t}$ has been described in the section above, the extracted MRoIs are also applicable to all the levels of scale space. Harris-Affine corners are detected as the initial interest points in the mth MRoI of different levels of $P_{G}^{t,l}$ and $P_{D}^{t,l}$ (2≤l≤L−1). Assume that $N_{m}$ initial interest points are detected. Because MRoIs may still include parts of the background and the torso, some noise points could be falsely detected, which need further filtering. On the one hand, the noise points in the background can be removed based on the adaptive depth constraint. On the other hand, the corners in the torso region need to be screened on the basis of grayscale and depth motion information to obtain accurate keypoints.

The greyscale and depth optical flows of an initial interest point $p(x_{l},y_{l})$ are calculated from $P_{G}^{t,l}$, $P_{G}^{t+1,l}$ and $P_{D}^{t,l}$, $P_{D}^{t+1,l}$ using the Lucas-Kanada algorithm [34]. If the depth and the optical flow of point p satisfy the adaptive constraints, Equation (7), p is deemed as a keypoint:

(7) $$\{\begin{array}{l} d_{t}^{p}\leq \theta \\ \left|v_{G}^{t,p}\right|=\sqrt{{\left(v_{G}^{t,r,p}\right)}^{2}+{\left(v_{G}^{t,c,p}\right)}^{2}}\geq \beta_{1}\\ \left|v_{D}^{t,p}\right|=\sqrt{{\left(v_{D}^{t,r,p}\right)}^{2}+{\left(v_{D}^{t,c,p}\right)}^{2}}\geq \beta_{2} \end{array},$$

where $v_{G}^{t,r,p}$ and $v_{G}^{t,c,p}$ are the horizontal and vertical velocities of point p, respectively, of the grayscale image; $v_{D}^{t,r,p}$ and $v_{D}^{t,c,p}$ are the horizontal and vertical velocities of point p, respectively, of the depth image; $\left|v_{G}^{t,p}\right|$ and $\left|v_{D}^{t,p}\right|$ are the velocity magnitudes of point p, respectively, of the grayscale and depth image; $d_{t}^{p}$ is the depth value of point p; θ is the local depth constraint threshold adaptively determined in the *m*th MRoI; $\beta_{1}$ and $\beta_{2}$, are, respectively, the local grayscale and depth motion constraint thresholds adaptively determined in the *m*th MRoI.

The purpose of determining θ is to adaptively search for the optimal threshold, which can accurately distinguish between large and small depth values. The purpose of determining $\beta_{1}$ and $\beta_{2}$ is to adaptively search for the optimal thresholds, which can accurately distinguish between high and low velocities. These processes are actually binarization, so θ, $\beta_{1}$ and $\beta_{2}$ can be determined using the Otsu algorithm in the *m*th MRoI.

**Figure 5:**
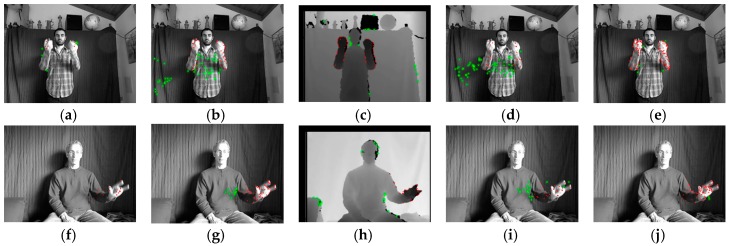
Keypoints detected by different spatiotemporal feature approaches. (**a**,**f**) The keypoints detected by 3D EMoSIFT; (**b**,**g**) The keypoints detected by 3D SMoSIFT4; (**c**,**h**) The keypoints detected by 3D SMoSIFT2; (**d**,**i**) The keypoints detected by MFSK; (**e**,**j**) The keypoints detected by the proposed approaches. Red points denote the keypoints detected from MBPs. Falsely detected keypoints are marked as green asterisks.

The extracted MRoIs, which have different depth and speed, can obtain their own local depth and motion constraints. For each MRoI, by its own depth and motion constraints, the initial interest points detected in the background and the torso regions are filtered well and in the MBP regions are retained as keypoints. Figure 5 shows the keypoints detected by 3D EMoSIFFT, 3D SMoSIFT4, 3D SMoSIFT2, MFSK and the proposed method. All keypoints detected at different levels of grayscale or depth scale space are mapped into the original grayscale image $G_{t}$ or depth frame $D_{t}$. Red points denote the keypoints detected from MBPs. Falsely detected keypoints are marked as green asterisks. In the second, third and fourth columns, a large number of noise points are falsely detected in the regions where shadow moving, depth value loss and clothes movements exist. That is because 3D SMoSIFT4, 3D SMoSIFT2 and MFSK detect keypoints from the whole grayscale images or depth frames according to empirical grayscale or depth motion constraints which are specified by constant values. On the contrary, only few noise points are detected in the last column. In the first column, there are also a small amount of noise points. That come out of 3D EMoSIFT using the grayscale optical flow and depth-dependent constraints simultaneously to detect keypoints. Although there are fewer noise points, the keypoints are sparse and nonuniform in the two hands and arms in Figure 5a. When compared with the proposed method, it is observed that the keypoints of 3D EMoSIFT, 3D SMoSIFT2/4 and MFSK methods are less dense than that of the proposed method, particularly for 3D EMoSIFT. Dense keypoints are essential to achieving better feature descriptors to represent gestures.

## 4. Feature Descriptor

The single or combined application of 3D SMoSIFT, HOG, HOF and MBH feature descriptors achieves excellent performance in one-shot learning gesture recognition, which has been demonstrated by some state-of-the-art approaches [1,9,10,11,35,36,37], and is also widely used for human activity recognition [15,30,38]. In this paper, 3D SMoSIFT, HOG, HOF and MBH feature descriptors are concatenated to represent gestures. Contrast with the 3D SMoSIFT and MFSK methods, the gradient space and motion space are extended to calculate these descriptors, which are useful for obtaining more representative RGB-D appearance and motion features of gestures.

Suppose that keypoint p is detected from the *l*th level of $P_{G}^{t}$ or $P_{D}^{t}$ (i.e., $P_{G}^{t,l}$ or $P_{D}^{t,l}$). To calculate the feature descriptors, eight local patches $\Gamma_{1}-\Gamma_{8}$ (32×32) around p are extracted. $\Gamma_{1}$ and $\Gamma_{2}$ are extracted from $P_{G}^{t,l}$ and $P_{D}^{t,l}$, respectively. $\Gamma_{3}$ and $\Gamma_{4}$ are extracted from $P_{G}^{t+1,l}$ and $P_{D}^{t+1,l}$, respectively. $\Gamma_{5}$, $\Gamma_{6}$, $\Gamma_{7}$ and $\Gamma_{8}$ in turn contain the grayscale horizontal, depth horizontal, grayscale vertical, and depth vertical velocity information. The Lucas-Kanada algorithm is adopted to calculate these grayscale and depth velocities from two pairs of local patches $\Gamma_{1}-\Gamma_{2}$ and $\Gamma_{3}-\Gamma_{4}$.

**Figure 6:**
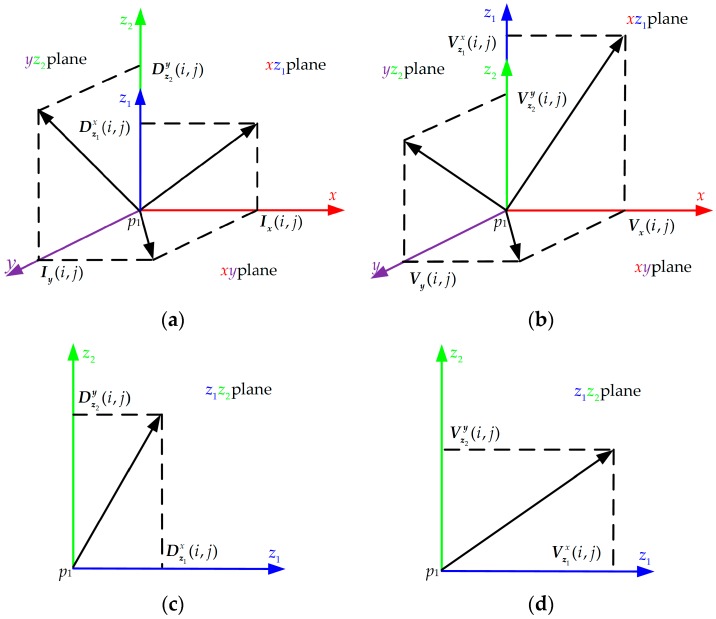
Extended gradient space and motion space. (**a**) 3D gradient space with xy, $xz_{1}$ and $yz_{2}$ planes; (**b**) 3D motion space with xy, $xz_{1}$ and $yz_{2}$ plane; (**c**) $z_{1}z_{2}$ plane made of $D_{z_{1}}^{x}(i,j)$ and $D_{z_{2}}^{y}(i,j)$; (**d**) $z_{1}z_{2}$ plane made of $V_{z_{1}}^{x}(i,j)$ and $V_{z_{2}}^{y}(i,j)$. (**a**,**c**) form the extended gradient space; (**b**,**d**) form the extended motion space.

As indicated in Figure 6a, 3D SMoSIFT constructs the 3D gradient space with three 2D gradient planes (xy, $xz_{1}$, $yz_{2}$ plane) based on $\Gamma_{1}-\Gamma_{2}$. For a neighborhood (32 × 32) point $p_{1}(i,j)$ of the keypoint p, its horizontal gradient $I_{x}(i,j)$ and vertical gradient $I_{y}(i,j)$ are calculated from $\Gamma_{1}$. $D_{z_{1}}^{x}(i,j)$ and $D_{z_{2}}^{y}(i,j)$ are the horizontal and vertical gradients of $p_{1}$ calculated from $\Gamma_{2}$. $I_{x}(i,j)$, $I_{y}(i,j)$, $D_{z_{1}}^{x}(i,j)$ and $D_{z_{2}}^{y}(i,j)$ are given as [1]:

(8) $$\begin{array}{l} I_{x}(i,j)=\Gamma_{1}(i,j+1)-\Gamma_{1}(i,j),\\ I_{y}(i,j)=\Gamma_{1}(i+1,j)-\Gamma_{1}(i,j),\\ D_{z_{1}}^{x}(i,j)=\Gamma_{2}(i,j+1)-\Gamma_{2}(i,j),\\ D_{z_{2}}^{y}(i,j)=\Gamma_{2}(i+1,j)-\Gamma_{2}(i,j). \end{array}$$

$I_{x}(i,j)$ and $I_{y}(i,j)$ form plane xy; $I_{x}(i,j)$ and $D_{z_{1}}^{x}(i,j)$ form plane $xz_{1}$; $I_{y}(i,j)$ and $D_{z_{2}}^{y}(i,j)$ form plane $yz_{2}$.

Three pairs of gradient magnitude and orientation of $p_{1}$ can be calculated in planes xy, $xz_{1}$ and $yz_{2}$, respectively. For all the neighborhood (32×32) points of keypoint p, we calculate their three pairs of gradient magnitude and orientation by the same method. Then, we generate three new patches $\Gamma_{xy}$, $\Gamma_{xz_{1}}$ and $\Gamma_{yz_{2}}$. The size of $\Gamma_{xy}$, $\Gamma_{xz_{1}}$ and $\Gamma_{yz_{2}}$ are 32×32. For each point with coordinate (i,j) in the patch $\Gamma_{xy}$, $\Gamma_{xz_{1}}$ or $\Gamma_{yz_{2}}$, it has two values: the gradient magnitude and orientation which are calculated in the plane xy, $xz_{1}$ or $yz_{2}$. Finally, a SIFT-like feature descriptor with 128 dimensions is calculated in each new patch. So the dimensionality of the feature descriptor vector, calculated in the 3D gradient space is 384 (128 × 3). On this foundation, we add a new plane $z_{1}z_{2}$, which is formed by $D_{z_{1}}^{x}(i,j)$ and $D_{z_{2}}^{y}(i,j)$. Using the above strategy, a new patch $\Gamma_{z_{1}z_{2}}$ is generated, as shown in Figure 6c. A SIFT-like feature descriptor, calculated from $\Gamma_{z_{1}z_{2}}$, also has 128 dimensions. Therefore, a 512-dimension (128×4) feature descriptor vector of keypoint p can be obtained from the extended gradient space with four planes (xy, $xz_{1}$, $yz_{2}$ and $z_{1}z_{2}$).

Figure 6b is the 3D motion space with three 2D gradient planes (xy, $xz_{1}$, $yz_{2}$ plane) based on $\Gamma_{5}-\Gamma_{8}$. $V_{x}(i,j)$, $V_{y}(i,j)$, $V_{z_{1}}^{x}(i,j)$ and $V_{z_{2}}^{y}(i,j)$ are given by $\Gamma_{5}(i,j)$, $\Gamma_{7}(i,j)$, $\Gamma_{6}(i,j)$ and $\Gamma_{8}(i,j)$ of neighborhood point $p_{1}(i,j)$. $V_{x}(i,j)$ and $V_{y}(i,j)$ form plane xy; $V_{x}(i,j)$ and $V_{z_{1}}^{x}(i,j)$ form plane $xz_{1}$; $V_{y}(i,j)$ and $V_{z_{2}}^{y}(i,j)$ form plane $yz_{2}$. 3D SMoSIFT uses the same method as the feature descriptor calculated in the 3D gradient space to get a 384-dimension descriptor vector in the 3D motion space. The same strategy continues to be adopted to extend the 3D motion space. A new plane $z_{1}z_{2}$ is formed by $V_{z_{1}}^{x}(i,j)$ and $V_{z_{2}}^{y}(i,j)$, as shown in Figure 6d; so, the extended motion space includes four planes (xy, $xz_{1}$, $yz_{2}$ and $z_{1}z_{2}$) as well. Thus, a feature descriptor vector with 512 (128×4) dimensions for keypoint p can be calculated in the extended motion space. Finally, these two feature descriptor vectors of keypoint p are concatenated into a vector with 1024 (512+512) dimensions. The extended 3D SMoSIFT feature descriptor contains more depth appearance and motion information to represent gesture.

The MFSK method calculates the HOG and HOF feature descriptors in $\Gamma_{1}$ and $\Gamma_{2}$ [9]. The local patch is divided into γ×γ cells. The gradient orientations are quantized into η bins [9]. The MFSK method calculates the grayscale and depth HOG descriptors in the xy and $z_{1}z_{2}$ planes of the extended gradient space. They can also be calculated in the $xz_{1}$ and $yz_{2}$ planes. The HOG feature descriptor vector, calculated in each plane, has γ×γ×η dimensions; thus, a HOG feature descriptor with 4×γ×γ×η dimensions is obtained. Similarly, a HOF feature descriptor can be computed from the extended motion space. The HOF descriptor vector also has 4×γ×γ×η dimensions.

Optical flow includes horizontal and vertical velocity components; so, there are two classes of MBH feature descriptors, i.e., MBHx and MBHy [20]. The MFSK method calculates the MBHx feature descriptors in patches $\Gamma_{5}-\Gamma_{6}$ and the MBHy feature descriptors in patches $\Gamma_{7}-\Gamma_{8}$. Different from the HOF feature descriptor, MBHx and MBHy represent the gradient of optical flow; so, they are calculated in the extended gradient space which has four planes (xy, $xz_{1}$, $yz_{2}$ and $z_{1}z_{2}$). For the MBHx feature descriptor, the extended gradient space is constructed in $\Gamma_{5}$ and $\Gamma_{6}$. $I_{x}(i,j)$, $I_{y}(i,j)$, $D_{z_{1}}^{x}(i,j)$ and $D_{z_{2}}^{y}(i,j)$ are calculated by Equation (8) where $\Gamma_{1}$ and $\Gamma_{2}$ are replaced by $\Gamma_{5}$ and $\Gamma_{6}$, respectively. Then, the MBHx descriptors are separately computed in the four planes, according to the calculation strategy of the original MBHx descriptor; so, each MBHx feature descriptor has 4×γ×γ×η dimensions, whereas the one calculated by the MFSK method has only 2×γ×γ×η dimensions. This is because the MFSK method calculates the MBHx descriptor in the xy and $z_{1}z_{2}$ planes only. Similarly, the MBHy feature descriptor with 4×γ×γ×η dimensions also can be calculated in $\Gamma_{7}$ and $\Gamma_{8}$ by the same method as the one used for the MBHy descriptor. The extended gradient space is also constructed by Equation (8) where $\Gamma_{7}$ and $\Gamma_{8}$ replace $\Gamma_{1}$ and $\Gamma_{2}$, respectively.

## 5. Special Case

Sometimes, when the motion speed of the user is very small in several frames, the user can be considered to be in a relatively static state at that moment. Relatively static frames do not contain useful motion features, and the appearance features of the human body cannot be extracted using the proposed method. When the motion speed of the user becomes larger, the last relatively static frame turns into the first motion frame. From this frame onwards, following the proposed method, not only the motion features of MBPs, but also the same appearance features as those of the relatively static frames can be extracted, so relatively static frames can be ignored. That does not result in loss of the motion and appearance features of gesture; instead, it improves the processing efficiency.

If $G_{t}$ is static relative to $G_{t+1}$, the grayscale information of the two consecutive images does not differ much. Hence, the grayscale correlation coefficient $\rho_{t,t+1}$ of $G_{t}$ and $G_{t+1}$ is larger than a predetermined threshold τ, so relatively static frames can be determined using the criterion $\rho_{t,t+1}\geq \tau$.

## 6. Experimental Results and Discussion

The proposed method are compared with the current state-of-the-art approaches using the 2011/2012 ChaLearn One-shot-learning Gesture Dataset (CGD) [21], the Cornell Activity Dataset-60(CAD-60) [39] and the MSRDailyActivity3D Dataset [40]. There is only one training sample per gesture category. Each training or test sample includes two videos, i.e., a grayscale video (converted from RGB video) and a depth video, both of which are captured by Kinect simultaneously. L=5, k=1.4 and σ=1.6 are used to construct the scale space [22,31].

To determine the appropriate dimension sizes for calculating feature descriptors, the effects of using different settings of γ and η on feature extraction are analyzed. The MLD scores are calculated with different values of γ∈[1,2,3,4] and η∈[2,4,8,16] on the development batch of the CGD (devel 01–devel 20). As shown in Table 1, the performance of the ALSTF features is relatively stable, and the best performance is 0.1240 when γ=3 and η=8. The MLD score 0.1263 of γ=2 and η=8 is the second best. Although its recognition performance is slightly worse, its descriptor (HOG + HOF + MBHx + MBHy) dimension 512 is less than half of that of the best one. Then we compare the computational efficiency of calculating the feature descriptors (HOG + HOF + MBHx + MBHy) on the two settings of γ and η. 47 pairs of videos (3926 grayscale and 3926 depth frames) are selected from the development batch to form a test set. Experiments are performed on a PC with C++ programs, CPU Intel^®^ Core™ i7-4700MQ @ 2.4 GHZ and RAM 8 GB. The average computation time of γ=3 and η=8 is about 119.4 ms/f. It is obviously slower than the second place setting, which is about 64.7 ms/f. Taking into account both the computational efficiency and the comparison with the MFSK approach using γ=2 and η=8, we adopt the trade-off with γ and η set to 2 and 8, respectively [9]. Consequently, the dimension of the HOG, HOF, MBHx or MBHy descriptors is 128 (4×2×2×8).

To determine the relatively static frames, based on trial-and-error, τ=0.995. The Bag of Word (BoW) model is adopted to represent gestures, and the parameter setting is the same as the one used in the literature [16]. The Nearest Neighbour (NN) classifier is used for classification and recognition.

### 6.1. Experiments on CGD Dataset

In the following experiments, four evaluation batches of CGD are used: development batch (devel 01–devel 20), validation batch (valid 01–valid 20), final batch (round1: final 01–final 20) and final batch (round 2: final 21–final 40). Each evaluation batch includes 20 sub-batches, and each sub-batch has 47 pairs of RGB and depth videos (10 fps, 320×240), which were captured by Kinect. Each pair of videos contains one to five gestures, therefore, there are 10 training gestures (corresponding to 10 classes of gestures) and 90 test gestures in every sub-batch. Every gesture has one training sample. For the sake of comparison, the Mean Levenshtein Distance (MLD) score [41], which was used by the challenge organizers, is adopted to evaluate the recognition performance. The recognition accuracy increases as the MLD score decreases, and vice versa.

#### 6.1.1. Evaluation of Keypoint Detection Approaches

We calculate the 3D MoSIFT, 3D EMoSIFT, 3D SMoSIFT and MFSK feature descriptors of keypoints detected by the proposed method, and compare them respectively with the 3D MoSIFT, 3D EMoSIFT, 3D SMoSIFT and MFSK spatiotemporal feature approaches on the validation and final (round 2) batches.

Table 2 shows that using the proposed keypoints, combined with each of the four feature descriptors, gets higher recognition accuracy than that of the corresponding spatiotemporal feature approach. The MLD scores decrease, on average, by 0.0313 and 0.0206 on these two batches. This is because, on the one hand, the proposed method minimizes the noise effect on the detection of accurate keypoints by adaptively extracting MRoIs, and on the other hand, the keypoints are detected by determining the adaptive local depth and motion constraints in each MRoI. Because of these reasons, the MBPs with speed differences can be covered by more spatiotemporal keypoints.

#### 6.1.2. Evaluation of Feature Descriptors

To evaluate the performance of the extended feature descriptors, the MFSK method is used in detecting the keypoints in the development batch (devel 01–devel 20) and in calculating the original (the same as [9]) and the extended 3D SMoSIFT, HOGHOF and MBH descriptors. It can be seen from Table 3 that, as compared with the original descriptors, the MLD scores 0.194, 0.187, and 0.181 of the extended descriptors decreased by 0.014, 0.011 and 0.007, respectively. Compared with the 3D SMoSIFT + HOGHOF + MBH descriptor, the accuracy of the extended 3D SMoSIFT + HOGHOF + MBH descriptor increases by 0.016. It is an improvement of about 10.3%. This is because the extended feature descriptors contain richer RGB-D appearance and motion information, which can represent gestures more fully.

#### 6.1.3. Comparison with Other Spatiotemporal Feature Approaches

This experiment further demonstrates the recognition performance of the proposed spatiotemporal feature on the final batch (round 2: final 21–final 40). It is compared with Cuboid, Harris 3D, Dense Trajectory, 3D MoSIFT, 3D EMoSIFT, 3D SMoSIFT and MFSK feature methods, and the results are shown in Table 4. Our method achieves the lowest score 0.0737, implying that its recognition accuracy is higher than that of the other state-of-the-art spatiotemporal feature approaches. In Table 4, RGB denotes that feature descriptors are calculated with the RGB data; RGB-D denotes that the RGB and depth data are simultaneously used to calculate the feature descriptors. The 3D MoSIFT-based, MFSK and the proposed features are originally designed to use the RGB-D data, so these features are not extracted from the RGB videos. The MLD scores of Cuboid, Harris 3D, 3D MoSIFT and 3D EMoSIFT features are derived from [6]. The MLD scores of the 3D SMoSIFT feature are derived from [1]. The MLD scores of Dense Trajectory and MFSK features are derived from [9].

For the Cuboid, Harris 3D and Dense Trajectory approaches, the descriptors calculated from the RGB-D data achieve higher recognition accuracy than that calculated from the RGB data alone. It is noteworthy that all the 3D MoSIFT-based methods, based on the RGB-D data, obtain the desired results. This, in effect, means that the use of RGB-D double channel data enhances the representation and discrimination capability of spatiotemporal feature.

The MLD scores of the proposed method, and also of the 3D MoSIFT-based and MFSK methods, are significantly lower than that of Cuboid or the Harris 3D feature approach. This is because the 3D MoSIFT-based, MFSK and the proposed method fuse the RGB and depth information well to represent the appearance and motion pattern of MBPs. It is difficult to extract distinctive appearance and motion pattern from only one RGB training sample for the Cuboid and Harris 3D approaches. When the RGB-D data is used, the recognition accuracy of the Cuboid and Harris 3D approaches improves, but they simply calculate the feature descriptors only in the RGB and depth channel and do not fuse the RGB-D information well. Therefore, their MLD scores are still relatively high. The Dense Trajectory feature approach, using only the RGB data, can get a respectable accuracy of 0.1470 which is comparable with that of the 3D MoSIFT and 3D EMoSIFT methods. This is closely related to the dense spatiotemporal features extracted by the Dense Trajectory approach. The dense features can overcome the effect of some noise, besides well representing the appearance and motion of the human body. However, the effect of noise still exists; so, even after using the RGB and depth data simultaneously, the MLD score 0.1365 of the Dense Trajectory approach is still higher than that of the 3D SMoSIFT, MFSK and the proposed method.

Additionally, the proposed approach should be categorized into the multi-feature-based gesture recognition approach. It is unfair to compare it with the single feature-based ones (i.e., Cuboid, Harris 3D, Dense Trajectory, 3D MoSIFT, 3D EMoSIFT, 3D SMoSIFT). So the MLD score of each adaptive feature approach (i.e., the extended 3D SMoSIFT, HOGHOF or MBH) is calculated on the final batch (round 2), as shown in the last three lines of the Table 4. The MLD scores of the extended 3D SMoSIFT, HOGHOF and feature approaches are 0.0833, 0.0953 and 0.1027, respectively, which are lower than the single feature-based approaches. That means the recognition accuracy of each adaptive feature approach is also superior to the single feature-based ones.

#### 6.1.4. Comparison with Other One-Shot Learning Gesture Recognition Approaches

The proposed method is compared using the validation batch (valid 01–valid 20) and the final batch (round 1: final 01–final 20) with five state-of-the-art one-shot learning gesture recognition approaches: mcHMM + LDA, HOG/HOF + DTW, 3D MoSIFT, HMM-based, motion signature analysis. The five teams corresponding to the above five approaches have made outstanding achievements in the ChaLearn Challenge. The experimental results are shown in Table 5.

The recognition accuracy of the motion signature analysis approach is far higher than that of other approaches. The team Alfine is ranked first in the two rounds of the challenge. Unfortunately, the details of their approach are not made public. Although there is a large gap in the MLD scores between our method and the motion signature analysis approach, the MLD scores 0.1069, 0.1156 achieved by our method are lower than those obtained by the other published approaches. The accuracies of the five state-of-the-art approaches are derived from [42].

mcHMM + LDA, HOG/HOF + DTW and HMM-based approaches employ HOG and HOF feature descriptors to represent gestures. Their keypoints are detected from the whole frame, and hence some noise points are falsely detected. The larger motion caused by noise affects the accuracy of the HOF descriptors too seriously to represent gesture motion. In addition, the HOG and HOF feature descriptors are not scale and rotation invariant. Using them alone to represent gesture features is unsuitable for one-shot leaning gesture recognition. So, their MLD scores 0.2084, 0.1702 are higher than those of the proposed method.

#### 6.1.5. MLD Score Analysis for Sub-Batches

**Figure 7:**
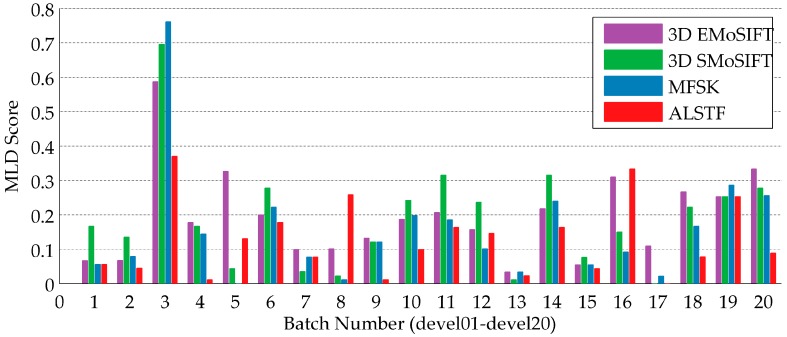
The results evaluated on sub-batches devel 01–devel 20 for 3D EMoSIFT, 3D SMoSIFT, MFSK and the proposed approaches.

In all of the above experiments, the MLD scores are computed at the batch level. To further verify the performance, the MLD scores of every sub-batch of development batch (devel 01–devel 20) are calculated using the 3D EMoSIFT, 3D SMoSIFT, MFSK methods and our method. The results are shown in Figure 7. On most of the sub-batches, the above methods achieve good recognition results. The MLD scores of the four approaches, on average, are 0.1943, 0.1881, 0.1552 and 0.1263. On devel 04, devel 09 and devel 13, only one or two gestures are falsely detected by our method. Especially on devel 17, all the test gestures are correctly recognized.

**Figure 8:**
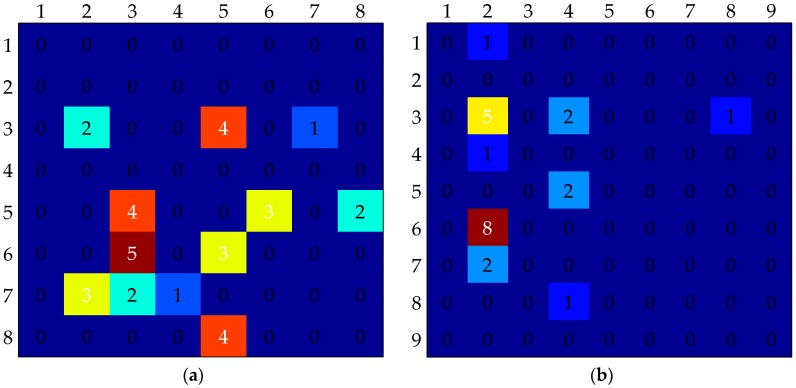
Confusion matrix of devel 03 and devel 19. (**a**) Confusion matrix of devel 03; (**b**) Confusion matrix of devel 19.

The MLD scores of devel 03 and devel 19 obtained by these four feature-based methods are higher. To find the reasons, the confusion matrices (Figure 8) are calculated using our method. Devel 03 includes eight training gestures and 92 test gestures. Devel 19 includes nine training gestures and 91 test gestures. It reveals that the gestures, which can easily be falsely recognized, have similar trajectories in the same sub-batch.

**Figure 9:**
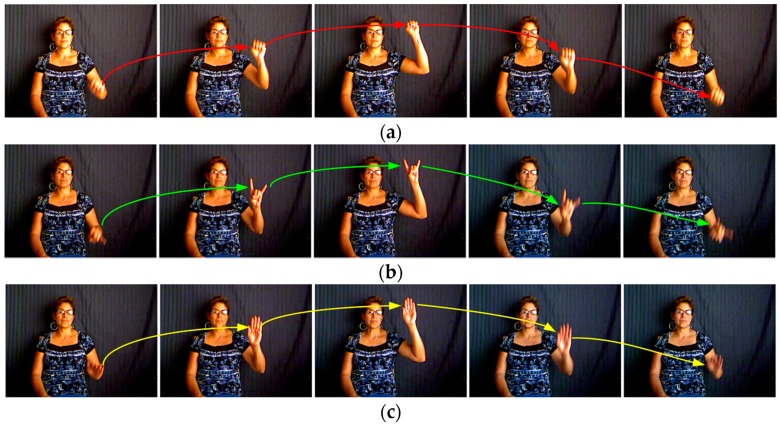
(**a**–**c**) Three gestures that have similar trajectories (color curves) but different hand shapes and appearances. Each column shows one stage of these gestures from the start frame to the last frame.

Their main differences are in the hand shapes and appearances, such as those shown in Figure 9. Feature descriptors calculated in the extended motion space contain the motion magnitude and orientation information of keypoints. We can consider that these feature descriptors represent the trajectory features of gestures. Since these four methods detect sparse keypoints, feature descriptors calculated in the gradient space could not well represent the shape and appearance of the hand when its region is large.

The overall recognition accuracy 0.1263 of the proposed method is better than that of the 3D EMoSIFT, 3D SMoSIFT and MFSK feature methods. This is because of relatively dense and accurate keypoints together with the corresponding extended feature descriptors, which are helpful to represent the motion and appearance features of gestures.

#### 6.1.6. Comparison of Computational Efficiency

We compare the computational efficiency of the proposed approach and related techniques. Forty seven pairs of videos (3926 grayscale and 3926 depth frames) are selected from the development batch to form a test set. The computational efficiency of 3D EMoSIFT, 3D SMoSIFT, MFSK and our methods are quantitatively compared using the test data. Experiments are performed on a PC with C++ programs, CPU Intel^®^ Core™ i7-4700MQ @ 2.4 GHZ and RAM 8 GB. As shown in Table 6, the computational efficiency of MFSK and our methods is higher than that of the 3D EMoSIFT method, but not as good as that of the 3D SMoSIFT method. Compared with the 3D SMoSIFT method, the addition of HOG, HOF and MBH feature descriptors leads to a decrease in computational cost of the MFSK method. Our method calculates the dense optical flow to extract MRoIs before detecting keypoints, and extends the dimension sizes of the feature descriptors, so it costs more time than the MFSK method. The unoptimized code also affects the computing efficiency of our method. If the code is optimized and a higher performance computer is used, our method may meet the requirement for real-time applications.

### 6.2. Evaluation on CAD-60 Dataset

CAD-60 includes RGB and aligned depth videos (30 fps, 640×480). It was captured using a Microsoft Kinect sensor in five different environments: office, kitchen, bedroom, bathroom, and living room. Three to four common activities are identified for each location, giving a total of twelve unique activities and several random activities [39]. The activities were performed by two males and two females. To test the proposed feature, we experimented with two test settings: leave-one-out cross validation setting and one-shot learning setting.

#### 6.2.1. Experiments in Leave-One-Out Cross Validation Setting

For leave-one-out cross validation setting, the model was trained on three of the four people, and tested on the fourth [39]. As can be seen in Table 7, the results of our method are comparable with those of the state-of-the-art. Our method obtains 89.7% precision, 86.1% recall and 87.86% F1 score. Although the results, on the whole, are not the best, they are comparable with other approaches. With the approaches described in [43,44,45], it is easier to locate the human’s moving parts and extract accurate features using skeleton information, so they achieve a higher accuracy than our method. Although the Kinect can effectively provide skeleton information, there are some cases where the skeleton cannot be extracted correctly and hence, cannot be extracted. Because our method does not need any preprocessing, it can still work in those cases.

#### 6.2.2. Experiment in One-Shot Learning Setting

In the other one-shot learning setting, only one sample per activity is used for training and the rest for testing. Further, random activities are ignored and only the rest of the twelve unique activities are used to evaluate the proposed feature.

**Figure 10:**
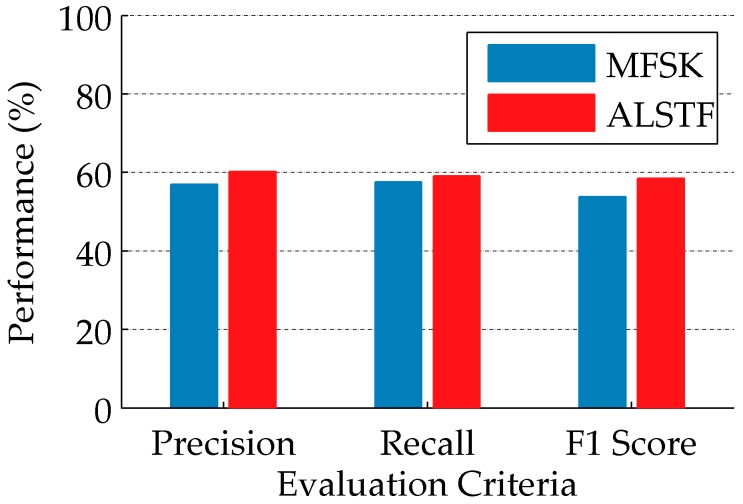
Performance comparison between MFSK and the proposed feature on the CAD-60 dataset in one-shot learning setting. Precision, recall and F1 score are used as accuracy measure.

The proposed feature is compared with the MFSK feature, as shown in Figure 10. The MFSK feature obtains the better accuracy compared with other published approaches on CGD Dataset. The proposed feature can obtain 60.2% precision, 59.1% recall and 58.51% F1 score, which are higher than the results of the MFSK feature: 57%, 57.6% and 53.8% [9].

### 6.3. Experiments on MSRDailyActivity3D Dataset

This dataset, which consists of 16 daily activities in the living room, was captured by a Kinect sensor. There are ten subjects and each subject performs one activity in two different poses: “sitting” and “standing”. So, the total number of the activity samples is 320 [40]. This dataset is particularly challenging, because many of the daily activities involve human-object interaction.

#### 6.3.1. Experiment in Leave-One-Out Cross Validation Setting

Table 8 shows the recognition accuracies of different state-of-the-art spatiotemporal feature approaches in leave-one-out cross validation setting. Our method achieves a recognition accuracy of 96.8%. This result is better than other approaches, considering the difficulties in this dataset.

#### 6.3.2. Experiment in One-Shot Learning Setting

**Figure 11:**
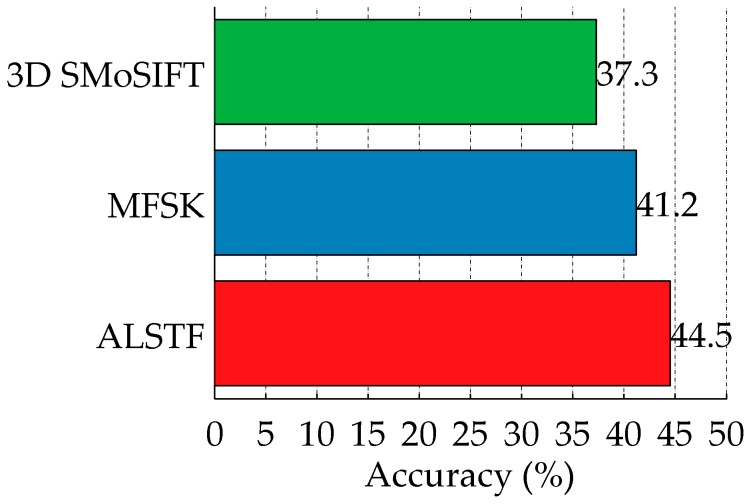
The comparison of 3D SMoSIFT, MFSK and the proposed spatiotemporal features on the MSR DailyActivity3D dataset in one-shot learning setting.

In one-shot learning setting, two samples (standing and sitting) of each activity are randomly selected as training samples. The recognition accuracy of 3D SMoSIFT, MFSK and the proposed methods are compared. The results are shown in Figure 11. Compared with the other two approaches, our method has significantly improved the recognition performance, achieving 44.5% accuracy. The accuracies have increased by 7.2% and 3.3%, respectively.

## 7. Conclusions

A distinctive adaptive local spatiotemporal feature has been developed to represent appearance and motion information of gesture for one-shot learning gesture recognition using RGB-D data. Adaptive extraction of MRoIs by utilizing the variance of grayscale and depth optical flows can minimize the effect of noise. MRoIs mainly include MBPs where sparse spatiotemporal features will be extracted. In each MRoI, Harris-Affine corners that satisfy adaptive local depth constraint, motion of grayscale and depth constraints are treated as keypoints. These keypoints, which are affine, scale, rotation and illumination-invariant, are accurately and sufficiently distributed in MBPs. The 3D SMoSIFT, HOG, HOF and MBH feature descriptors are calculated in the extended grayscale and motion spaces to represent the rich appearance and motion features of gesture.

The experimental results on the CGD dataset show that the proposed feature can outperform other state-of-the-art spatiotemporal feature-based approaches and the published one-shot learning approaches. It also obtains respectable results on the CAD-60 and MSRDailyActivity3D datasets under both leave-one-out cross validation setting and one-shot learning setting.

Future work, based on the results of this paper, will be focused on accurately detecting the hand region to obtain more hand shape and appearance features. This will be beneficial for improving the performance in recognizing gestures which have similar trajectories but different hand shapes. We need to study motion estimation in dynamic 3D scene [51,52], which is to enable the robot to recognize gestures in motion. We also plan to design a real-time HRI system for practical application.

## Figures and Tables

**Table 1:** The analysis of using some different settings of γ and η to calculate feature descriptors. The values in the brackets are feature descriptor (HOG + HOF + MBHx + MBHy) dimensions.

	*η*	2	4	8	16
*γ*					
1		0.1564 (32)	0.1484 (64)	0.1394 (128)	0.1420 (256)
2		0.1382 (128)	0.1439 (256)	0.1263 (512)	0.1343 (1024)
3		0.1393 (288)	0.1384 (576)	0.1240 (1152)	0.1413 (2304)
4		0.1337 (512)	0.1305 (1024)	0.1459 (2048)	0.1486 (4096)

**Table 2:** The comparison of keypoint detection methods on the validation batch (valid 01–valid 20) and final batch (round 2: final 21–final 40).

Approach	MLD Score	
	Valid 01–Valid 20	Final 01–Final 20
3D MoSIFT keypoint + 3D MoSIFT descriptor [42]	0.1824	0.1448
3D EMoSIFT keypoint + 3D EMoSIFT descriptor [6]	0.1595	0.1331
3D SMoSIFT keypoint + 3D SMoSIFT descriptor [1]	0.1740	0.1130
MFSK keypoint + MFSK descriptor [9]	0.1270	0.0925
Proposed keypoint + 3D MoSIFT descriptor	0.1412	0.1221
Proposed keypoint + 3D EMoSIFT descriptor	0.1345	0.1127
Proposed keypoint + 3D SMoSIFT descriptor	0.1294	0.0879
Proposed keypoint + MFSK descriptor	0.1125	0.0783

**Table 3:** The performance validation of the extended 3D SMoSIFT, HOGHOF and MBH feature descriptors (devel 01–devel 20).

Descriptor	MLD Score (Devel 01–20)
MBH [9]	0.208
HOGHOF [9]	0.198
3D SMoSIFT [9]	0.188
3D SMoSIFT + HOGHOF + MBH	0.155
Extended MBH	0.194
Extended HOGHOF	0.187
Extended 3D SMoSIFT	0.181
Extended 3D SMoSIFT + HOGHOF + MBH	0.139

**Table 4:** The performance comparison of different spatiotemporal feature approaches on the final batch (round 2: final 21–final 40).

Spatiotemporal Feature	MLD Score (Final 21–Final 40)	
	RGB	RGB-D
Cuboid	0.3139	0.2806
Harris 3D + HOG	0.2346	0.2268
Harris 3D + HOF	0.2906	0.2712
Harris 3D + HOGHOF	0.1886	0.1819
Dense Trajectory	0.1470	0.1365
3D MoSIFT	-	0.1448
3D EMoSIFT	-	0.1331
3D SMoSIFT	-	0.1130
MFSK	-	0.0925
Proposed feature (ALSTF)	-	0.0737
Proposed keypoint + Extended MBH		0.1027
Proposed keypoint + Extended HOGHOF		0.0953
Proposed keypoint + Extended 3D SMoSIFT		0.0833

**Table 5:** The performance comparison with 5 state-of-the-art one-shot learning gesture recognition approaches on the valid and final (round 1: final 01–final 20) batches.

Approach/Team	MLD Score	
	Vaild 01–Valid 20	Final 01–Final 20
mcHMM + LDA/Immortals	0.2488	0.1847
HOGHOF + DTW/Turtle Tamers	0.2084	0.1702
3D MoSIFT/Joewan	0.1824	0.1680
HMM-based/Pennect	0.1797	0.1652
Motion signature analysis/Alfine	0.0995	0.0734
Proposed feature (ALSTF)	0.1069	0.1156

**Table 6:** The computational efficiency of 3D EMoSIFT, 3D SMoSIFT, MFSK and the proposed feature.

Approach	Average Time (ms/f)
3D EMoSIFT	646.4
3D SMoSIFT	45.3
MFSK	119.1
Proposed feature (ALSTF)	163.7

**Table 7:** The performance comparison of several state-of-the-art approaches on the CAD-60 dataset under leave-one-out cross validation setting.

Approach	Accuracy (%)		
	Precision	Recall	F1 Score
Hierarchical MEMM [39]	67.9	55.5	61.08
3D SMoSIFT [1]	74.8	65.8	70.01
Ni et al., 2013 [46]	75.9	69.5	72.56
Guptal et al., 2013 [47]	78.1	75.4	76.73
Zhang and Tian 2012 [48]	86.0	84.0	84.99
MFSK 2016 [9]	87.1	83.8	85.42
Zhu et al., 2014 [43]	93.2	84.6	88.69
Parisi et al., 2015 [44]	91.9	90.2	91.47
DBMM [45]	91.1	91.9	91.50
Proposed feature (ALSTF)	89.7	86.1	87.86

**Table 8:** The comparison of different spatiotemporal features on the MSR DailyActivity3D dataset in leave-one-out cross validation setting.

Spatiotemporal Feature	Accuracy (%)
3D MoSIFT	75.9
Hon4d [49]	80.0
RGGP [50]	85.6
Fourier temporal pyramid feature + Actionlet ensemble model [40]	85.8
3D EMoSIFT	86.0
3D SMoSIFT	93.2
MFSK	95.7
Proposed feature (ALSTF)	96.8
